# Mechanisms of Moral Disengagement Used to Justify School Violence in Sicilian Primary School

**DOI:** 10.3390/ejihpe10030050

**Published:** 2020-06-29

**Authors:** Inmaculada Méndez, Giuseppa Liccardi, Cecilia Ruiz-Esteban

**Affiliations:** Department of Evolutionary Developmental and Educational Psychology, University of Murcia, 30100 Murcia, Spain; inmamendez@um.es (I.M.); g.liccardi@um.es (G.L.)

**Keywords:** school climate, school violence, moral disengagement, emotional management, peer relationships

## Abstract

This study investigated the mechanisms of moral disengagement most commonly used to justify school violence in Sicilian primary school. The main objective of this study was to analyze the mechanisms of moral disengagement that are set in motion by those involved in situations of school violence (victims, aggressors, and bystanders) in Sicilian primary school. Likewise, the differences by gender and age are investigated. A total of 113 subjects in primary school were recruited (56.6% girls). The ages ranged from 8 to 11 (*M* = 9.56, *SD* = 0.99). The first scale used was the Bullying Inventory by Olweus (1993) in the Italian translation by Genta, Menesini, Fonzi, Costabile, and Smith (1996) and the questionnaire on moral disengagement developed by Caprara, Barbaranelli, Vicino, and Bandura (1996) is also used. The regression analysis showed that the sociodemographic variables and the mechanisms of moral disengagement are different depending on a person’s role (aggressor, victim, or bystander). Moral justification predicted the role of victim in school violence, dehumanization predicted the role of the aggressor (and gender), and the disclosure of responsibility (and dehumanization) predicted the role of the bystander in school violence. The conclusions of this study will facilitate the prevention of school violence, for example, by promoting social integration and minimizing situations of school violence (emphasizing morality, ethics, etc.), thereby establishing balanced and satisfactory interpersonal relationships.

## 1. Introduction

Moral disengagement was defined by Bandura in his studies, theory, and developed instruments [1,2]. Before violent or transgressive behavior, there is a partial or total deactivation of the control system that internally regulates moral conduct into acceptable conduct, making violent behavior possible. According to Bandura, the self-regulation system of moral conduct is invariable and does not work continuously. Thus, it can be selectively deactivated by engaging with the relevant cognitive mechanisms. According to Bandura [1,2], there are eight mechanisms of moral disengagement: moral justification (used to demonstrate that violent conduct is performed for socially valid and moral purposes), euphemistic language (the subject uses euphemistic terms to justify violent actions and reduce personal responsibility), advantageous comparison (the subject wants to demonstrate that there are facts more serious than the violent act committed), displacement of responsibility (the transfer of responsibility, where the subject shifts the blame for his or her violent actions towards authorities or actors that legitimize violence), disclosure of responsibility (the subject who has carried out violent actions seeks to spread guilt in a group), distorted consequences (the subject seeks to minimize the damage caused), blame attribution (the subject seeks justify his or her violent conduct via the victim’s conduct), and dehumanization (lowering the humanity of the subject to the condition of an animal or object). In this way, the effects of the reprehensible act are minimized, and the subject maintains his or her moral principles and criteria, thereby, avoiding sanctions or experiencing moral conflict.

Moral disengagement mechanisms are used by both boys and girls interchangeably and increase with age [1,3]. The 13-year-old bullies presented higher levels of moral disengagement compared to the group of younger schoolchildren (9 years) [3].

The meta-analysis of Gini, Pozzoli, and Hymel [4] showed that moral disengagement is a significant correlate of aggressive behavior among children and youths. The appearance of each different mechanism is diffused, and the emergence of these differences is not independent of their use. That is, to a greater or lesser extent, these mechanisms help explain the global concept of moral disconnection, but no clear factors explain the preferential use of one mechanism over another [5]. The first studies carried out with schoolchildren in the Italian population [3,5,6,7,8,9] demonstrate that aggressor males present higher levels of cognitive mechanisms of moral disengagement than victims, provocative victims (a victim who has decided to respond to aggression actively), or girls. Victims have the lowest levels of moral disconnection, and bystanders maintain medium levels compared to those directly involved. The level of moral disengagement was found to increase with age [3]. Likewise, different behaviors appear in different cultural contexts [3,10].

School violence is among the manifestations of aggressive behavior between schoolchildren [11,12]. This includes aggressive behaviors that occur between an individual or a group of people against a victim who is unable to defend him or herself—that is, there is an imbalance of power. This imbalance can manifest itself through physical violence, verbal violence, and social exclusion, as well as through new technologies—to harass other peers, using the internet and mobile phones—(e.g., cyberbullying). This is a worldwide phenomenon. A UNESCO report [13] showed that Italy has achieved a significant decrease in its prevalence of bullying because the country has also invested in research and evaluation. The two main school-based programs (No Trap! and KiVA) were effective in achieving sustained reductions in school violence in Italian schools. At least one in three students (32%) have been bullied by their peers at school at least once in the last month. Cyberbullying affects one in ten children. In Europe and North America, bullying of a psychological type is the most frequent.

Therefore, the main objective of this study was to analyze the mechanisms of moral disengagement that are set in motion by those involved in situations of school violence (victims, aggressors, and bystanders) in Sicilian primary school. Likewise, the secondary objective was to investigate the differences by gender and age. Among the hypotheses of our study, we hope to find that the use of moral disengagement mechanisms increases with age as well as among aggressors. We also hope to find the sociodemographic variables (gender and age) and the use of moral disengagement mechanisms that predict the roles involved in situations of school violence (victims, aggressors, and bystanders).

## 2. Materials and Methods

### 2.1. Participants

In the school, there were a total of 348 in primary school and 113 subjects were recruited (56.6% girls and 43.4% boys). The participants were aged from 8 to 11 years (*M* = 9.56, *SD* = 0.99) and were in the third, fourth, or fifth grade. The age breakdown of the participants is as follows: 17.7% (*n* = 20) were 8 years old, 27.4% (*n* = 31) were 9 years old, 36.3% (*n* = 41) were 10 years old, and 18.6% (*n* = 21) were 11 years old; 0.9% had repeated an academic year. In total, 34 students were not included in the study because they did not have written parental consent or were absent from school on the day of the study. A total of 1.8% were born outside of Italy; 10.7% of fathers and 21.6% of mothers were born outside of Italy (see Table 1). For this study, data from a Sicilian educational center were randomly selected (there are two schools in the whole city).

### 2.2. Instruments

The first scale was the Bullying Inventory designed by Olweus [14] in the Italian translation by Genta, Menesini, Fonzi, Costabile, and Smith [15]. This questionnaire measures the frequency of having been attacked, having attacked, and having observed situations of physical aggression, verbal aggression, indirect aggression, cyberbullying, etc. It also evaluates sociodemographic data, including gender (male/female) and age. A subscale is obtained for the aggressor, another for the victim, and another for the bystander (From 1 = never to 5 = several times a week). Cronbach’s alpha values α = 0.80 are very acceptable, ranging from 0.80 to 0.90 in several studies carried out by Olweus and collaborators [11]. In our study, this value was α = 0.80 for this instrument (an example item: "I had rumors spread about me”).

Afterward, the questionnaire about moral disengagement by Caprara, Barbaranelli, Vicino, and Bandura [16] was used. The scale has 32 items and consists of eight factors for mechanisms of moral disengagement according to Bandura: moral justification, euphemistic language, advantageous comparison, displacement of responsibility, disclosure of responsibility, distorted consequences, blame attribution, and dehumanization (from 1 = never to 4 = always; an example item: “It is alright to fight to protect your friends”). In different studies, the estimated Cronbach´s alpha value was around 0.82 [16,17,18]. The reliability of the subscales was measured to have a Cronbach´s alpha of 0.81.

### 2.3. Procedure

After receiving approval from the Ethics Committee and approval from the directors of the educational centers, parental authorization and informed consent from the students were requested. The instruments were administered during a 50-min session in the classrooms of the students with the supervision of the researcher to clarify any doubts (for example, leave blank if you do not know how to answer, type of mark to answer, mark a single answer, etc.). In each classroom, there were 16 or so students. The questionnaires were in the Italian version, as it is easy to understand. They were anonymous, self-administered, and confidential questionnaires.

### 2.4. Data Analysis

A cross-sectional design was followed for this study. Descriptive techniques such as frequencies, percentages, means, and standard deviations were used. The Pearson correlation was used to analyze the relationships between the continuous variables and to determine the differences in each of the variables independently (age and mechanisms of moral disengagement). Student’s *t*-test was used for the independent samples. Hierarchical regression analyses were used to contrast the predictive power of the groups of independent variables (socio-demographic variables such as age and gender in model 1 and the mechanisms of moral disengagement in model 2) in relation to the dependent variable under study (victim, aggressor, or bystander), as well as the relations between those variables and the “enter” method. All analyses were performed with SPSS 24.0 (IBM, Armonk, NY, USA).

### 2.5. Ethics Approval

The protocol for this study was approved by the Ethics Committee for Clinical Investigations of the University of Murcia in November 2019 (ID:2353/2019), according to the approved guidelines and the declaration of Helsinki with written informed consent from all participants (parents and their children).

## 3. Results

Table 2 shows the types of school violence according to the victim or the aggressor. Table 3 shows Bystander behavior and attitude towards bullying. In the sociodemographic variables, no significant differences were found in the Pearson correlation between age and the role of victim, aggressor, or bystander. The results of the Student’s *t*-test did not show significant mean differences between the mechanisms of moral performance and gender for the aggressor and victim. However, for the bystanders, significant mean differences appeared, with the females’ mean (*M* = 15.23; *SD* = 3.68) higher than that of the males (*M* = 13.43; *SD* = 5.01), that is, there were more boys than girls among the bystanders (See Table 4).

Regarding the mechanisms of moral disengagement, according to the sociodemographic variables for age, a significant positive correlation was only found between age and disclosure of responsibility (*rp* = 0.330, *p* = 0.000). The results of the Student’s *t*-test did not show significant mean differences between the mechanisms of moral performance and gender (see Table 5).

On the other hand, for the relationship between school violence and the mechanisms of moral disengagement, using the aggressor as the criterion and sociodemographic variables (gender and age) (in model 1) alongside the mechanisms of moral disengagement (in model 2) as the predictor in the regression analysis (see Table 6). The standardized beta regression coefficient showed that, among all the predictor variables, dehumanization (*Beta* = 0.411; *t* = 4.009; *p* = 0.000) was significant.

In addition, using the victim as the criterion and sociodemographic variables (gender and age) (in model 1) alongside the mechanisms of moral disengagement (model 2) as the predictor in the regression analysis (see Table 7). The standardized beta regression coefficient showed that, among all the predictor variables, moral justification (*Beta* = 0.245; *t* = 2.300; *p* = 0.023) was significant.

Finally, using the bystander as the criterion and sociodemographic variables (gender and age) (in model 1) along with the mechanisms of moral disengagement (model 2) as predictor variables in the regression analysis (see Table 8). The standardized Beta regression coefficient showed that, among all the predictor variables, gender (*Beta* = 0.200; *t* = 2.238; *p* = 0.027), disclosure of responsibility (*Beta* = 0.321; *t* = 3.073; *p* = 0.003), and dehumanization (*Beta* = 0.219; *t* = 2.211; *p* = 0.029) were significant.

## 4. Discussion

Among the most frequent forms of school violence, the victims highlighted being excluded from their groups of friends, having their personal belongings stolen or damaged, having their physical appearance mocked, being bullied with false rumors, and receiving threats. The aggressors highlighted excluding the victims from their groups of friends. The results of the study show that, in school violence, there are no differences by age or gender between the aggressors and victims. However, in our study, girls were more often bystanders than boys.

The moral disengagement mechanism was used by both boys and girls interchangeably [1]. For this reason, prevention or intervention strategies should be directed at all schoolchildren, without differentiating by gender. A significant positive correlation was only found between age and disclosure of responsibility.

On the other hand, the regression analysis shows that 17.9% of the variance of the aggressor in school violence can be explained by dehumanization. Our hypothesis is that dehumanization predicts being an aggressor in school violence. The use of the moral disengagement mechanism among the aggressors may be explained by a lack of empathy, a real absence of feelings of guilt, and, therefore, an emotional state of coldness. In the same way, the aggressors tried to justify their actions to experience positive feelings and self-approval before their own negative behavior, thus making them more egocentric [3,8]. In other words, an emotionally disconnected aggressor uses his or her arguments to justify moral disengagement, avoid the emotional effects that self-blame may cause, and seek self-approval [1,2].

In our study, moral justification predicted the role of the victim in school violence. The fact that victims have less moral disengagement than aggressors is usually because they have a greater sense of personal moral responsibility in situations of school violence and may even understand the arguments of moral disconnection used by the aggressor [1,2,3,7].

Finally, in our study, gender, disclosure of responsibility, and dehumanization predicted the role of bystanders in school violence. The bystanders still participate in school violence since they are aware of the existence of such situations. Bystanders have intermediate scores between the aggressor and the victim. This suggests that they have a passive role in maintaining harassment since they understand the situations. They do not seek solutions, they do not denounce the aggressors, and they do not support the victims [3,6,9].

Our study has shown that moral perceptions are mediators of judgments about events of school violence. The moral disengagement mechanism provides strategies through which immoral actions are made to seem moral, thus creating a lack of connection between reasoning and moral behavior. The self-regulation system of moral conduct is invariable and does not work continuously. Therefore, it can be selectively deactivated by activating the relevant cognitive mechanisms. Since moral emotions are self-conscious, the role of behavioral regulation is fundamental and can promote the performance of actions perceived as morally good or inhibit behaviors and attitudes that are evaluated as bad or harmful [1,2,3]. In our study, bystanders who were not involved in bullying assumed different roles: non-involved bystanders, defenders, accomplices, and boosters [19]. For fear of confronting the aggressor, some students engage morally in false rules of silence [3].

Thus, both victims and aggressors present difficulties in managing their emotional lives in the face of significant moral transgressions. For victims, it is necessary to promote emotional regulation and coping strategies. Aggressors usually show little sensitivity towards the pain of others; they are proud, insensitive, and cold, with a lack of empathy and little guilt for their attacks against their victims. They present knowledge of social norms and values α = 0.80 but make use of mechanisms of egocentric moral disengagement from situations. In the case of the victims, they have high moral sensitivity to the suffering of others, which makes them feel guilty and ashamed when they are involved as victims. Such situations create insecurity and feelings of incompetence, producing guilt and shame [3,10]. Emotions of indifference or disconnection from damage usually emerge, which can increase suffering in such situations [20]. Therefore, it is necessary to develop empathy as a necessary link to prosocial and moral behavior [21]. It is also possible to promote educational programs that encourage physical activities, such as fighting to cope with aggressiveness [22]. Likewise, it is necessary to equip bystanders with the skills to report known situations of harassment [3].

Finally, Ferrara, Ianniello, Villani, and Corsello [23] note that in Italy, law no. 71/17 (the "anti-cyberbullying law") provides legal protection to victims of cyberbullying. Thus, all schools have the responsibility to educate their students on the risks of the internet.

It is necessary to carry out programs to mitigate the consequences of bullying and cyberbullying on the quality of life and health of schoolchildren. In this area, the UNESCO report [13] shows that programs like No Trap! (begun in 2008) and KiVA (begun in 2013) are effective in achieving sustained reductions in school violence in Italian schools. The KiVa anti-bullying program confirms the relevance of temperament as a moderator in such an intervention (focused actions on bystanders and teacher training) [24]. The Tabby Improved Prevention and Intervention Program (TIPIP) for cyberbullying and cyber victimization decreased both cyberbullying and cyber victimization among students who received the intervention (components of the project: teachers, parents, in class activities, and online materials) [25]. Likewise, the NoTrap! anti-bullying program was effective in reducing bullying and victimization (increasing defending behavior only in the voluntary recruitment condition and a training module for teachers was implemented too) [26]. It is very important that teachers are trained in bullying and consider students with special educational needs (for example, sensory disability, communication and language difficulties, etc.) [2].

As the limitations of our study, it should be noted that this is a cross-sectional study focused on a single age bracket and, above all, that the questionnaires may be affected by social desirability [3]. It would be appropriate to develop longitudinal studies that provide more information [27]. Moreover, the variance explained in the regression analyses is not high, so it is necessary to investigate other influencing variables. Thus, research that integrates moral behavior from a comprehensive standpoint that includes emotions, motivations, cognition, attitudes, and behaviors remains necessary, especially regarding moral internalization and prosocial behavior [28,29]. More cross-cultural studies and investigations should also be performed at an early age [30]; including the relevance of temperament [20]. Likewise, the meta-analyses reviewed by Gini et al. [4] indicate that it is necessary to carry out a study of contextual factors, the influences of development, etc. Researchers could also study the types of teacher responses to situations of bullying as moral disengagement strategies could be influenced by teacher responses in situations of bullying and victimization [31].

## 5. Conclusions

Bullying and cyberbullying situations occur in school. School violence is a relational phenomenon that relies on context. Thus, it is necessary to work with victims, aggressors, bystanders, family, and teachers to solve both bullying and cyberbullying. Both bullying and cyberbullying cause health-related and psychological symptoms [11,12]. Two consequences of bullying are maladjustment and greater involvement in criminal behavior at the adolescent stage [31,32,33].

Students involved in situations of school aggression engage in the most frequent moral disengagement arguments. Thus, prevention at primary school is necessary [34]. Consequently, we must promote social integration and minimize situations of school violence. This could entail a socialization process in which the norms and their transgressions are clear while considering ever-evolving situations (emphasizing morality, ethics, social skills, etc.), thereby establishing balanced and satisfactory interpersonal relationships [3].

## Figures and Tables

**Table 1:** Country of birth of the scholar and the parents.

Variable		Frequency	Percentage
Country of birth	Italian	111	99.2%
	Outside of Italy	2	1.8%
Father’s country of birth	Italian	101	89.4%
	Outside of Italy	12	10.6%
Mother’s country of birth	Italian	89	78.8%
	Outside of Italy	24	21.2%

**Table 2:** Types of school violence according to victim or aggressor.

Types	None	Once or Twice	2 or 3 Times a Month	Once a Week	Several Times a Week
I have been bullied in this type:					
Physical aggression	102 (90.3%)	8 (7.1%)	3 (2.7%)		
Threats	94 (83.2%)	14 (12.4%)	3 (2.7%)	1 (0.9%)	1 (0.9%)
Theft	79 (69.9%)	16 (14.4%)	14 (12.4%)	3 (2.7%)	1 (0.9%)
Race or Color	109 (96.5%)	2 (1.8%)	1 (0.9%)	1 (0.9%)	
Physical appearance	82 (72.6%)	16 (14.4%)	12 (10.6%)	1 (0.9%)	2 (1.8%)
Difficulty or disability	104 (92%)	5 (4.4%)	2 (1.8%)	1 (0.9%)	1 (0.9%)
Sexual abuse	105 (92.9%)	5 (4.4%)	2 (1.8%)	1 (0.9%)	
Insults	98 (86.7%)	9 (8%)	5 (4.4%)	1 (0.9%)	
Excluded from group of friends	79 (69.9%)	21 (18.6%)	10 (8.8%)	2 (1.8%)	1 (0.9%)
False rumors	94 (83.2%)	13 (11.5%)	5 (4.4%)		1 (0.9%)
Cyberbullying	109 (96.5%)		2 (1.8%)		2 (1.8%)
Others	105 (92.9%)	5 (4.4%)	1 (0.9%)	1 (0.9%)	1 (0.9%)
I have bullied in this type:					
Physical aggression	110 (97.3%)	2 (1.8%)	1 (0.9%)		
Threats	108 (95.6%)	4 (3.5%)	1 (0.9%)		
Theft	110 (97.3%)	3 (2.7%)			
Race or color	111 (98.2%)	1 (0.9%)	1 (0.9%)		
Physical appearance	104 (92%)	6 (5.3%)	1 (0.9%)		2 (1.8%)
Difficulty or disability	10 (95.6%)	4 (3.5%)		1 (0.9%)	
Sexual abuse	109 (96.5%)	3 (2.7%)		1 (0.9%)	
Insults	104 (92%)	9 (8%)			
Excluded from group of friends	94 (83.2%)	17 (15%)	1 (0.9%)		1 (0.9%)
False rumors	107 (94%)	4 (3.5%)	2 (1.8%)		
Cyberbullying	110 (97.3%)	2 (1.8%)		1 (0.9%)	
Others	110 (97.3%)	3 (2.7%)			

**Table 3:** Bystander behavior and attitude towards bullying.

Types	Never	Sometimes	Many Times	Everyday
When I see bullying:				
They defend the victim	30 (26.5%)	49 (43.4%)	21 (18.6%)	13 (11.5%)
They laugh, they support the aggressors	89 (78.8%)	17 (15%)	5 (4.4%)	2 (1.8%)
They leave the victims alone	86 (76.1%)	21 (18.6%)	5 (4.4%)	1 (0.9%)
They don’t pretend anything	76 (67.3%)	21 (18.6%)	15 (13.3%)	1 (0.9%)
They abandon the bullies	45 (39.8%)	45 (39.8%)	15 (13.3%)	8 (7.1%)
They notify the teacher	28 (24.8%)	31 (27.4%)	27 (23.9%)	27 (23.9%)
They support the victim in moments of calm	26 (23%)	36 (31.9%)	23 (20.4%)	28 (24.8%)
Try to join the bullies	63 (55.8%)	28 (24.8%)	12 (10.6%)	10 (8.8%)

**Table 4:** Differences by gender for roles in school violence.

Roles	Male M(SD)	Female M(SD)	*t*	*p*
Victim	14.92 (3.98)	14.63 (3.03)	0.445	n.s.
Aggressor	12.73 (2.43)	12.84 (2.91)	−0.212	n.s.
Bystander	13.43 (5.01)	15.23 (3.68)	−2.214	0.037

Note: n.s. not significant.

**Table 5:** Differences by gender for mechanisms of moral disengagement.

Mechanisms of Moral Disengagement	Male M(SD)	Female M(SD)	*t*	*p*
Moral justification	7.49 (1.91)	7.55 (1.99)	−0.155	n.s.
Euphemistic language	5.19 (1.72)	4.94 (1.31)	0.835	n.s.
Advantageous comparison	5.35 (1.65)	5.53 (1.76)	−0.571	n.s.
Displacement of responsibility	6.23 (2.30)	7.16 (2.83)	1.803	n.s.
Disclosure of responsibility	7.82 (2.44)	7.47 (2.34)	0.765	n.s.
Distorted consequences	6.02 (2.04)	6.17 (2.14)	−0.381	n.s.
Blame attribution	5.55 (1.89)	5.77 (1.78)	−0.613	n.s.
Dehumanization	5.04 (1.62)	5.05 (1.39)	−0.021	n.s.

Note: n.s. not significant.

**Table 6:** Hierarchical regression analyses to contrast the predictive power of the groups of independent variables in relation to the aggressor.

Model	R²	Adjusted r-squared	R² Change	F Change	F
1	0.001	−0.017	0.001	0.065	0.065
2	0.179	0.098	0.177	2.753 **	2.217 *

Notes. * *p* ≤ 0.05, ** *p* ≤ 0.01.

**Table 7:** Hierarchical regression analyses to contrast the predictive power of the groups of independent variables in relation to the victim.

Model	R²	Adjusted r-squared	R² Change	F Change	F
1	0.002	−0.016	0.002	0.100	0.100
2	0.162	0.080	0.160	2.440 *	1.974 *

Notes. * *p* ≤ 0.05.

**Table 8:** Hierarchical regression analyses to contrast the predictive power of the groups of independent variables in relation to the bystander.

Model	R²	Adjusted r-squared	R² Change	F Change	F
1	0.043	0.025	0.043	2.454	2.454
2	0.234	0.159	0.192	3.190 **	3.121 **

Notes. ** *p* ≤ 0.01.
